# The Fundamental Neutron Physics Beamline at the Spallation Neutron Source

**DOI:** 10.6028/jres.110.015

**Published:** 2005-06-01

**Authors:** Geoffrey Greene, Vince Cianciolo, Paul Koehler, Richard Allen, William Michael Snow, Paul Huffman, Chris Gould, David Bowman, Martin Cooper, John Doyle

**Affiliations:** University of Tennessee, Knoxville, TN; Oak Ridge National Laboratory, Oak Ridge, TN; Indiana University, Bloomington, IN; North Carolina State University, Raleigh, NC; Los Alamos National Laboratory, Los Alamos, NM; Harvard University, Cambridge, MA

**Keywords:** fundamental neutron physics, neutron source, spallation neutron source

## Abstract

The Spallation Neutron Source (SNS), currently under construction at Oak Ridge National Laboratory with an anticipated start-up in early 2006, will provide the most intense pulsed beams of cold neutrons in the world. At a projected power of 1.4 MW, the time averaged fluxes and fluences of the SNS will approach those of high flux reactors. One of the flight paths on the cold, coupled moderator will be devoted to fundamental neutron physics. The fundamental neutron physics beamline is anticipated to include two beam-lines; a broad band cold beam, and a monochromatic beam of 0.89 nm neutrons for ultracold neutron (UCN) experiments. The fundamental neutron physics beamline will be operated as a user facility with experiment selection based on a peer reviewed proposal process. An initial program of five experiments in neutron decay, hadronic weak interaction and time reversal symmetry violation have been proposed.

## 1. Introduction

Cold neutrons and ultracold neutrons have been employed in a wide variety of investigations that shed light on important issues in nuclear, particle, and astrophysics in the determination of fundamental constants and in the study of fundamental symmetry violation. In many cases, these experiments provide information not available from existing accelerator-based nuclear physics facilities or high-energy accelerators. Until very recently, most of the research in this area has been based at cold sources at reactors. Such sources provide intense continuous beams of cold and ultracold neutrons.

The Spallation Neutron Source (SNS) offers an extraordinary opportunity for fundamental neutron physics (FNP). In the past, measurements in this field have been significantly limited by statistical and systematic effects. The SNS offers significant gains in both areas. It will have, by far, the highest peak neutron source intensity in the world. The proposed beam will be the most intense pulsed beam in the world for fundamental neutron physics. The fact that the SNS will be a pulsed source offers profound advantages for the reduction of systematic effects. The time-averaged neutron fluence from our proposed beamline at the SNS will be greater than that at any continuous neutron source in the United States. When the SNS reaches its final design goal of ≈2 MW, the flux and fluence will be within a factor of ≈3 to 4 times that at the highest flux beam at the Institut Laue-Langevin (ILL) [1].

Very significantly, the SNS, as a new facility, provides an exceptional opportunity to fully optimize the design of the beamlines. This is especially important in the reduction of backgrounds and the minimization of magnetic interference, which have proven to be serious problems at other neutron facilities.

In general, the advantages of the SNS in reducing systematic errors lie in four main areas:

1. Utilizing the time structure of the beam to analyze background and to separate the signal from parasitic effects that have different velocity dependence (*important for the experiments that study the weak nucleon-nucleon (NN) interaction via gamma asymmetry measurements and neutron spin rotation*).
2. Utilizing the time structure of the beam to make both precise and accurate determinations of neutron beam polarization with polarized ^3^He gas cells (*important for the beta asymmetry measurements of the A and B correlation coefficients in neutron decay*).
3. Utilizing developments in neutron guide technology, particularly curved “benders” to transport the beam far away from other equipment and experiments without significant loss of flux, thereby reducing gamma-ray and neutron backgrounds. The proposed external UCN facility will be far from other instruments.
4. Finally, the design of an independent external experimental facility allows the opportunity to address seismic/vibration noise that is particularly important for some experiments with UCNs.

A number of specific experiments have been identified as being particularly well suited to a cold-neutron program at the SNS. These are identified elsewhere in these proceedings and include the precise measurement of the neutron lifetime using magnetically trapped UCNs, the determination of the gamma-ray asymmetry in the capture of polarized neutrons on light nuclei, the precise measurement of a complete set of beta asymmetry parameters in polarized neutron decay, the determination of the parity non-conserving neutron spin rotation in light nuclei (H and ^4^He), and the search for a nonzero neutron electric dipole moment (EDM), using UCNs and superfluid helium.

## 2. The Fundamental Neutron Physics Beamline at the SNS

**Fig. 1:**
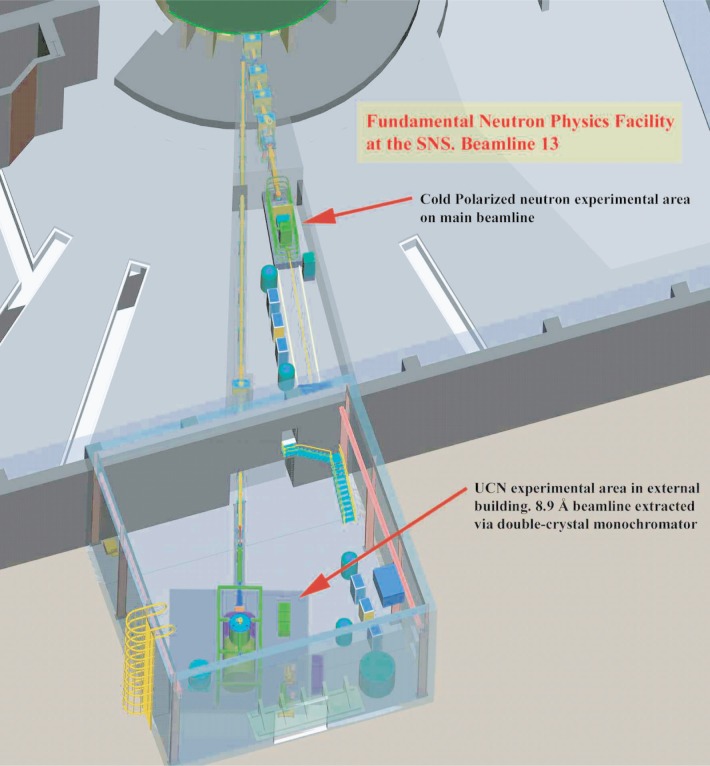
The fundamental neutron physics beamline at the SNS showing the cold neutron area inside the SNS target building and the external UCN facility. For scale, the existing n + p → d + γ apparatus is shown in at the cold beam position, and the proposed neutron EDM apparatus is shown in the external facility.

The FNP beamline facility will be located on neutron flight path 13 (FP13) at the SNS (Fig 1). The FNP beamline will consist of neutron guides, choppers, secondary shutters, monochromators, and shielding along with the necessary utilities, safety and radiation protection equipment, and appropriate ancillary equipment. One guide will terminate at approximately 15 m from the cold moderator, inside the SNS target building. It will be used for measurements that require a cold-neutron beam with a broad wavelength distribution. A second guide will transmit the neutrons to an external experimental facility adjacent to the SNS target building. This facility will be used for experiments that require ultracold neutrons.

The fundamental neutron physics beamline “views” a “partially-coupled” liquid hydrogen moderator. This moderator has the highest cold flux of the SNS moderators. The exit face of the moderator possesses a flat portion of 10 cm × 12 cm area. This moderator is viewed by a 10 cm × 12 cm, *m* = 3.5 supermirror guide. The guide penetrates the core vessel insert region and extends to within ≈0.9 m of the moderator face. At a distance of ≈2.2 m from the moderator is the primary beam shutter, which extends 1.8 m along the beam. A section of curved neutron bender extends through this shutter and is designed to precisely align with the static guide elements on both sides when the shutter is open. Between the main shutter and the end of the guide are 4 frame-overlap choppers. A secondary shutter will be placed downstream of the last chopper and will serve as the local experimental shutter.

The beamline for the UCN station begins with the double-crystal monochromator that diffracts a monochromatic neutron beam centered at 0.89 nm out of the primary polychromatic beam. The first crystal will be located between the first and second frame-definition choppers. The first chopper will be configured to have a “notch” such that 0.89 nm neutrons are passed for all operating conditions. This effectively decouples the operation of the polychromatic and 0.89 nm beamlines. The beam is diffracted horizontally using alkali-intercalated graphite crystals. The mosaic spread of this crystal can be made large enough (≈2° full width at half maximum) to match the neutron guide divergence so that all of the neutrons that can efficiently produce UCNs UCNs in ^4^He by one-phonon down scattering are reflec-reflected. The once reflected neutrons are incident on a second alkali monochromator that reflects the neutrons towards the external facility. The phase space distribution of the 0.89 nm neutrons is approximately returned to the distribution before reflection from the first crystal and thus can be efficiently guided to the experimental station with an *m* = 3.5 guide. The UCN beam-line ends at ≈40 m from the moderator in a shielded enclosure of dimensions approximately 15 m × 13 m × 10 m. This external building includes a vibrationally isolated floor slab for support of the experiments, cryogenic service, and road access for experimental equipment.

## 3. Project Status

The fundamental Neutron Physics Beamline is currently under construction at Oak Ridge National Laboratory. The cold neutron beamline is planned for completion in early 2008 and the ultracold neutron beamline is scheduled for completion in 2010. Both beamlines will be operated as user facilities with experimental approval and beam allocation based on a proposal driven, peer review system.
